# The “phosphorus pyramid”: a visual tool for dietary phosphate management in dialysis and CKD patients

**DOI:** 10.1186/1471-2369-16-9

**Published:** 2015-01-20

**Authors:** Claudia D’Alessandro, Giorgina B Piccoli, Adamasco Cupisti

**Affiliations:** Department of Experimental and Clinical Medicine, University of Pisa, Pisa, Italy; Department of Clinical and Biological Sciences, ASOU San Luigi, University of Turin, Turin, Italy

**Keywords:** Phosphorus, Diet, Food pyramid, dietary counselling, CKD, ESRD, Education, renal diseases

## Abstract

Phosphorus retention plays a pivotal role in the onset of mineral and bone disorders (MBD) in chronic kidney disease (CKD). Phosphorus retention commonly occurs as a result of net intestinal absorption exceeding renal excretion or dialysis removal. The dietary phosphorus load is crucial since the early stages of CKD, throughout the whole course of the disease, up to dialysis-dependent end-stage renal disease.

Agreement exits regarding the need for dietary phosphate control, but it is quite challenging in the real-life setting. Effective strategies to control dietary phosphorus intake include restricting phosphorus-rich foods, preferring phosphorus sourced from plant origin, boiling as the preferred cooking procedure and avoiding foods with phosphorus-containing additives. Nutritional education is crucial in this regard.

Based on the existing literature, we developed the “phosphorus pyramid”, namely a novel, visual, user-friendly tool for the nutritional education of patients and health-care professionals. The pyramid consists of six levels in which foods are arranged on the basis of their phosphorus content, phosphorus to protein ratio and phosphorus bioavailability. Each has a colored edge (from green to red) that corresponds to recommended intake frequency, ranging from “unrestricted” to “avoid as much as possible”.

The aim of the phosphorus pyramid is to support dietary counseling in order to reduce the phosphorus load, a crucial aspect of integrated CKD-MBD management.

## Introduction: the importance of phosphorus restriction

Phosphorus restriction is a mainstay of the nutritional treatment of mineral and bone disorders in chronic kidney disease (CKD-MBD). CKD-MBD is a clinical and physiopathological component that dramatically influences survival and quality of life of renal patients, with significant impact on health care costs [1]. Changes in calcium, calcitriol, PTH and FGF-23 are highly prevalent, but increased serum phosphorus levels is the main trigger for CKD-MBD [2]. In CKD patients, phosphorus retention occurs as a result of net intestinal absorption exceeding renal excretion and/or dialysis removal. This highlights the role of the diet in controlling the effective phosphorus load, starting from the early CKD stages.

Phosphorus is ingested both as a natural component and as a food additive.

As a natural food component, phosphorus is available as inorganic phosphate salts or as constituent of phosphoproteins, membrane phospholipids, ATP, ADP, DNA, RNA. On average, about 60% of dietary phosphorus is absorbed in the intestine as inorganic phosphorus, reaching up to 80% in the presence of high calcitriol levels, while the bioavailability of phosphorus of plant-origin, namely phytates, is very low (<40%) [3].

Conversely, the net gastrointestinal absorption of phosphorus is maximal (approaching 100%) for phosphate salts added as food preservatives [3].

Unfortunately, dietary phosphorus control is quite challenging in the real-life setting.

Therefore, information, education and counseling are needed to effectively integrate dietary interventions into the therapeutic approach of CKD-MBD.

## Which strategies may reduce dietary phosphorus intake?

### Dietary protein restriction

The restriction of protein intake in non-dialysis CKD patients is generally associated with a lower phosphorus intake. The direct relationship between protein and phosphorus dietary content is well known: on average, a mixed diet contains 12–14 mg of phosphorus per gram of protein [4, 5]. During the “conservative” management of advanced CKD, the use of protein-restricted diets facilitates the reduction of dietary phosphorus intake. Conversely, phosphorus restriction is hard to achieve on dialysis given the high protein requirements: hence, dialysis patients may benefit from other strategies, as described below.

### Increasing the intake of foods with low phosphorus content and/or low phosphorus bio-availability

An analysis of phosphorus content (mg/100 g edible part) in the various food groups shows that the highest load comes from nuts, hard cheeses, egg yolk, meat, poultry and fish. Reporting the phosphorus content as mg per gram of protein (mg/g protein) is especially useful for identifying which foods supply less phosphorus with the same amount of protein. Based on the relationship between phosphorus and proteins, we assumed an upper limit of 12 mg/g to identify foods with a “favorable” phosphorus to protein ratio [6].

Besides the absolute content, a crucial point is the net intestinal absorption of phosphorus. In general, intestinal absorption is lower for phosphorus of plant origin than for phosphorus of animal origin, such as from meat, fish, poultry and dairy products [7, 8].

As previously mentioned, added phosphorus is almost completely absorbed: phosphoric acid is usually added to soft drinks (cola-drinks in particular) [7]. There is large variability in type and content of phosphorus-containing preservatives, depending on the manufacturer: for example, most orange and lemon sodas do not contain phosphorus-based additives, but phosphoric acid is added to some brands.

### Boiling foods

Boiling causes demineralization of food, thus reducing phosphorus as well as sodium, potassium, and calcium content in both vegetable and animal-derived products. The degree of mineral loss is proportional to the amount of boiling water that is used, the size of the pieces, the cooking time and the absence of the peel for plants. Jones et al. reported a phosphorus reduction of 51% for vegetables, 48% for legumes, and 38% for meat after boiling [9].

It is noteworthy that boiling reduces the phosphorus content with a negligible loss of nitrogen [10], leading to a more favorable phosphorus to protein ratio.

### Identifying and avoiding phosphate additives

Phosphorus is the main component of several additives (phosphoric acid, phosphates and polyphosphates) used in industrial food processing to extend conservation, enhance color or flavor, and retain moisture. Food preservatives are added during the various stages of production, processing, preparation, packing, transport or storage [11]. Inorganic phosphorus salts are almost completely absorbed [12].

The amount of phosphorus from preservatives is considerable when compared to the natural phosphorus content.

Current regulations require reporting the presence of phosphorus-containing additives on the food labels, but specifying the amounts is not required and is not available in most food composition databases. Furthermore, in Europe, food labeling reports the preservatives either by their full name (as in the USA) or with an abbreviation (as the “E” series): for instance from E340 to E349 are phosphorus-containing preservatives used as antioxidants and acidity regulators, while from E450 to E458 serve as thickeners, emulsifiers and regulators. Hence, this extra phosphorus is sometimes also called “hidden phosphorus” since it does not usually appear in the common databases and food compositions tables [11, 13]. In a recent study, Leon et al. estimated that the extra burden of phosphorus coming from processed food may reach 700–800 mg per day [14, 15]. Such a high content may impair the effects and increase the costs of phosphate binder therapy which is expected to remove no more than 200–300 mg of phosphorus per day [16]. Sullivan et al. showed that 3 months of educational intervention on how to avoid foods with phosphorus-containing additives contributes to an average reduction of 1 mg/dl in serum phosphorus levels [17].

## Nutritional counseling

Patient information and education play a key role in nutritional care management. Renal patients need education and information about dietary sources of phosphorus and especially about the so-called hidden phosphorus. CKD patients are often unaware of artificially added phosphorus in food and drinks [18]. Knowledge about phosphorus is overall lower than knowledge about other nutrients (namely sodium, potassium and proteins) as assessed by a 25-item CKD nutritional knowledge assessment tool (CKDKAT-N) [19, 20]. A similar gap in knowledge has also been reported for health care professionals [20].

A systematic review of the educational strategies for phosphorus reduction in CKD patients with hyperphosphatemia showed an average reduction of serum phosphorus of 0.72 mg/dL after any educational intervention; the reduction increased to 1.07 mg/dL when educational interventions lasted over 4 months [21].

Assessment and control of dietary phosphorus intake is a complex, difficult task. Emerging educational initiatives include food labeling using a “traffic light” scheme, motivational interviewing techniques, and the “Phosphate Education Program” that aims at steering patients towards the correct use of phosphorus binders [22, 23].

In any case, a multidisciplinary approach is required. The team should include nephrologists, renal dietitians and nurses, but should be focused on the direct involvement of patients and care-givers, with particular attention to the family members involved in food selection, purchase and preparation.

The renal dietitian plays a pivotal role: a dietitian is “someone who puts the doctor’s prescription in the pot”, as we like to say in our daily clinical practice: his/her intervention should not be limited to telling the patient what to avoid, but should provide solutions and suggest alternative choices, especially when making the dietary plan.

How the dietitian interacts with the patients is as important as the dietary prescription: an understanding and non judging relationship is crucial for the patient to successfully adhere to the suggestions, thus sustaining the efforts to change habits in order to fulfill the dietary prescriptions [24, 25]. Furthermore, many brochures for CKD patients designed to facilitate their food choices [26, 27] are available also on-line. Although we strongly believe that every nephrology unit should have a dietitian, this goal is not widely attained: in this cases, the availability of simple but effective tools may be very important for a nurse-led educational programs for phosphorus lowering.

## Food pyramids

The food pyramid is a visual tool widely used in nutritional education strategies [28–31]. Several versions of food pyramids have been around since the late 70s. The first food pyramid was published in Sweden in 1974 [32], while an official U.S. Department of Agriculture version, targeted to the general population, was published in 1992 [31, 33–36]. The main goals of the food pyramids included supporting nutrient adequacy and moderation through pictures focused on the variety as well as on the proportion of energy, added fats and sugar content.

Several updates and alternatives followed: at present, over 25 Countries and Organizations have published adapted food pyramids [37–43]. The new versions are even more image-based so as to be more impressive and understandable.

One well known example is the Mediterranean diet pyramid, which graphically highlights the food groups to be consumed daily, weekly or less frequently: the graphic presentation is considered an important contribution to the worldwide popularity of the Mediterranean diet [44]. The Mediterranean diet pyramid has been subsequently modified to reflect the dietary changes occurring within the Mediterranean societies. The new graphic representation was conceived as a simplified, main-frame pyramid to be adapted to the various countries (i.e., portion sizes) and to the various geographical, socio-economic and cultural contexts of the Mediterranean area [45].

In January 2013, a Decree by the Italian Ministry of Health promoted the development of an Italian food pyramid for a healthy lifestyle. Two main versions have been produced: a weekly food pyramid, based on food group distribution during the week, and a daily pyramid consisting of six levels in which food groups are arranged in increasing order to highlight the different nutrients and energy content.

**Figure 1:**
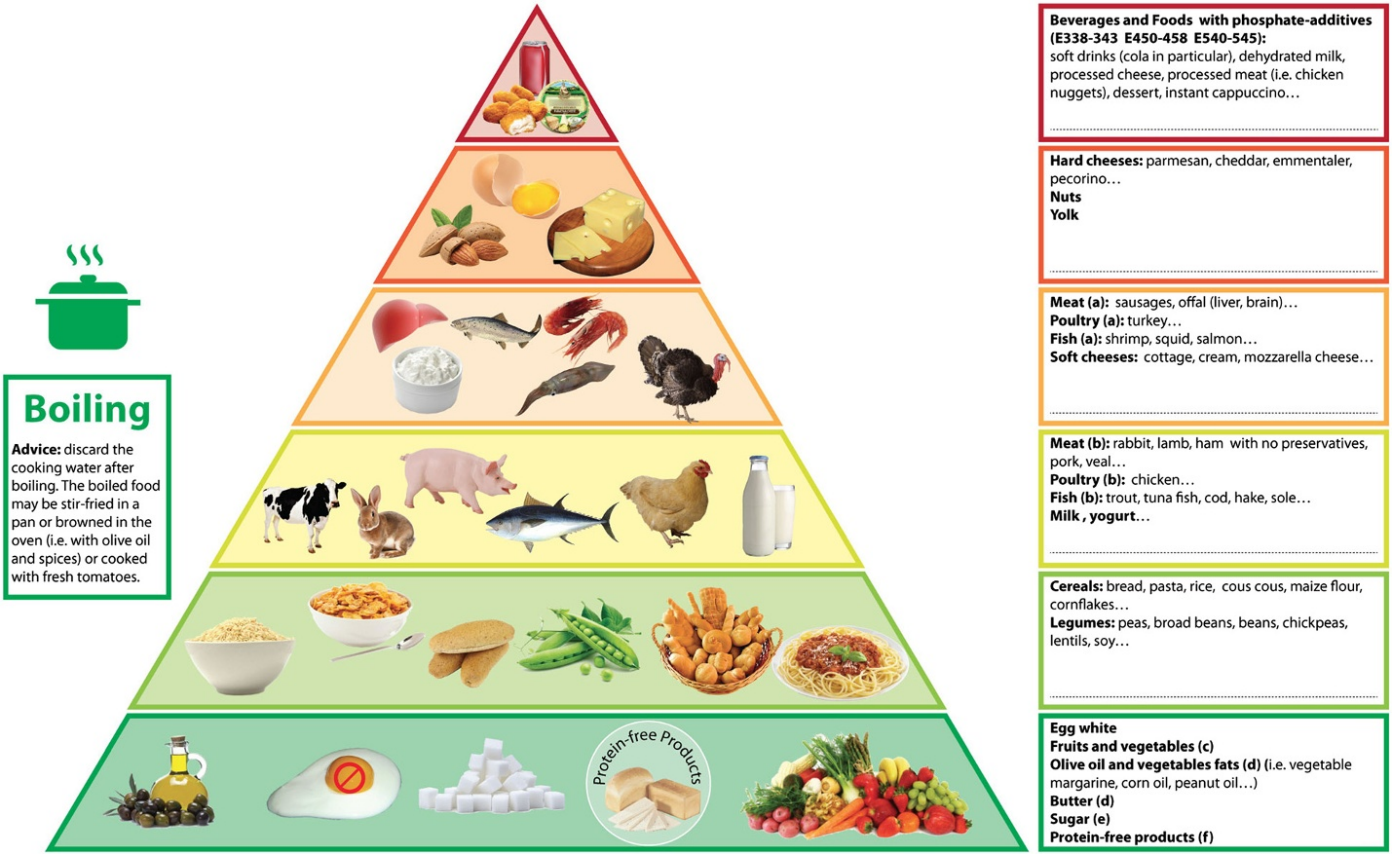
**The phosphorus pyramid.** Foods are distributed on six levels on the basis of their phosphorus content, phosphorus to protein ratio and phosphorus bioavailability. Each level has a colored edge (from green to red, through yellow and orange) that corresponds to recommended consumption frequency, which is the highest at the base (unrestricted intake) and the lowest at the top (avoid as much as possible). a) foods with unfavorable phosphorus to protein ratio (>12 mg/g); b) foods with favorable phosphorus to protein ratio (<12 mg/g); c) fruits and vegetables must be used with caution in dialysis patients to avoid excessive potassium load; d) Fats must be limited in overweight/obese patients, to avoid excessive energy intake; e) sugar must be avoided in diabetic or obese patients; f) protein-free products are dedicated to patients not on dialysis therapy and who need protein restriction but a high energy intake.

This is the context in which we developed our “Phosphorus Pyramid” (Figure 1).

## The phosphorus pyramid

The Phosphorus Pyramid is a visual tool that was designed to present the phosphate load of various foods. It was built upon the current nutritional databases and the existing literature on dietary phosphorus content, bio-availability, and processing (Figure 1) [4, 9–11, 13, 40].

The objective is to help the viewer to identify which foods cause a lower or a higher effective phosphate load: the distribution of food on the various floors should support the choice without the need to memorize the phosphorus content of each food item.

The pyramid consists of six floors in which foods are arranged on the basis of phosphorus content, phosphorus to protein ratio and phosphorus bioavailability. Each level has a colored edge (from green to red) that corresponds to recommended intake frequency.

At the base of the pyramid, the first level (green edge) contains foods with very low phosphorus content (i.e., sugar, olive oil, protein-free foods) or very low bio-available phosphorus (i.e., fruit and vegetables). It also includes white-egg which has an extremely favorable phosphorus to protein ratio and is a source of proteins with high biological value and no cholesterol, all of which are issues of particular importance especially in dialysis patients [46]. The intake of these foods is unrestricted. However, during counseling the operator should address special warnings to: *diabetic patients*, who should avoid sugar and not exceeding with fruit consumption ; *overweight or obese patients*, who should reduce sugar, olive oil, vegetables fats and butter intake; *dialysis patients*, who should limit fruits and vegetables consumption to avoid excessive potassium intake. In this regard the suggestions given in the green box regarding cooking may be useful also to reduce potassium intake from vegetables.

Finally, the use of protein-free products is targeted to CKD patients not on dialysis therapy who need protein restriction but a high energy intake.

The second level mainly includes vegetable foods, richer in phosphorus but mainly as phytate, hence with less intestinal absorption: cereals (white bread, pasta, rice, cornflakes) or legumes (peas, broad beans, soy). The suggested intake is 2–3 servings per day.

The third level includes foods of animal origin: lamb, rabbit, ham or fish such as trout, tuna fish, cod, hake, sole are indicated on account of their relatively low phosphorus to protein ratio.

A special warning regards farmed fish, as they are usually fed with flour and preparations rich in bioavailable phosphorus to promote rapid growth, resulting in sharply increasing the phosphorus content in the edible parts. Milk and yogurt are also in this section: they have high phosphorus content but one portion a day does not significantly influence the total amount of dietary phosphorus. The suggested intake is no more than 1 serving per day.

The fourth level shows foods with higher phosphorus to protein ratio. These include various products such as turkey, offal (liver, brain) and shrimp, squid, salmon, and soft cheeses. The suggested intake is one serving per week.

The fifth level contains foods with very high phosphorus content such as nuts, yolk and hard cheeses. The suggested intake is no more than 2–3 serving per month.

The top of the pyramid, the sixth level, includes foods with phosphorus-containing additives (cola beverages, processed meat, processed cheese), which should be avoided as much as possible.

The boxes on the right provide further information and are intended to allow the pyramid to be tailored to the individual patient: namely, specific foods may be added (in the empty spaces in the boxes on the right) during counseling, based on phosphorus content, phosphorus to protein ratio, and bio availability [6–8].

The boiling pot on the left side suggests boiling as the best cooking method to reduce the phosphorus content. The box provides the suggestion that boiled food can be simmered with olive oil, garlic and parsley, browned in the oven with olive oil and spices, or cooked with fresh tomatoes to improve taste and appearance.

## Summary

Dietary phosphorus restriction is a “leitmotiv” throughout the various CKD stages.

A simple and effective approach towards reducing the dietary phosphorus intake without affecting adequate protein intake consists in avoiding foods which are high in phosphorus or that contain phosphorus additives, preferring foods with lower phosphorus to protein ratio, and boiling as the preferred initial cooking method. Dietary counseling can lead to better control of phosphorus intake.

The phosphorus pyramid herein proposed is an original, visual, user-friendly tool for nutritional education. It can support patients and caregivers in making the right food choices by encouraging adherence to dietary prescriptions, which is the crucial component for CKD-MBD.

Validation studies are needed to assess the yield of this tool and to improve and adapt it to different clinical and socio-economic settings.
